# The human papillomavirus type 16 L1 protein directly interacts with E2 and enhances E2-dependent replication and transcription activation

**DOI:** 10.1099/vir.0.000162

**Published:** 2015-08

**Authors:** Abida Siddiqa, Karen Campos Léon, Claire D. James, Muhammad Faraz Bhatti, Sally Roberts, Joanna L. Parish

**Affiliations:** ^1^​ School of Cancer Sciences, College of Medical and Dental Sciences, University of Birmingham, Birmingham B15 2TT, UK; ^2^​ Atta-ur-Rahman School of Applied Biosciences (ASAB), National University of Sciences and Technology (NUST), Sector H-12, Kashmir Highway, Islamabad 44000, Pakistan

## Abstract

The human papillomavirus (HPV) E2 protein is a multifunctional protein essential for the control of virus gene expression, genome replication and persistence. E2 is expressed throughout the differentiation-dependent virus life cycle and is functionally regulated by association with multiple viral and cellular proteins. Here, we show for the first time to our knowledge that HPV16 E2 directly associates with the major capsid protein L1, independently of other viral or cellular proteins. We have mapped the L1 binding region within E2 and show that the *α*-2 helices within the E2 DNA-binding domain mediate L1 interaction. Using cell-based assays, we show that co-expression of L1 and E2 results in enhanced transcription and virus origin-dependent DNA replication. Upon co-expression in keratinocytes, L1 reduces nucleolar association of E2 protein, and when co-expressed with E1 and E2, L1 is partially recruited to viral replication factories. Furthermore, co-distribution of E2 and L1 was detected in the nuclei of upper suprabasal cells in stratified epithelia of HPV16 genome-containing primary human keratinocytes. Taken together, our findings suggest that the interaction between E2 and L1 is important for the regulation of E2 function during the late events of the HPV life cycle.

## Introduction

Human papillomaviruses (HPVs) are small, double-stranded DNA viruses that infect epithelia in multiple areas of the body, causing benign warts or, in some cases, cancer. Infection is established in the undifferentiated basal layer of the epithelia, and expression of the early genes E6, E7, E1 and E2 is initiated from the early promoter (P97 in HPV16). The combined expression of the E6 and E7 oncoproteins delays cell cycle exit and cellular differentiation, which are necessary for replication and amplification of viral genomes (Doorbar *et al.*, 2012). The E2 protein is a specific DNA-binding protein that is important for the replication of HPV genomes, by recruiting the viral helicase E1 to the origin of replication (Ori) (Sanders & Stenlund, 2001). In addition, E2 controls transcription of the early genes by association with the transcriptional enhancer immediately upstream of the early promoter (Bernard *et al.*, 1989; Kovelman *et al.*, 1996).

The E2 protein is folded into three distinct domains; the N-terminal transactivation domain, which is separated from the C-terminal DNA-binding and dimerization domain (DBD) by a disordered and flexible hinge region (Giri & Yaniv, 1988). E2 does not possess enzymic activity, but by association with a plethora of viral and cellular proteins, co-ordinates virus genome replication, transcriptional regulation, viral genome partitioning and cell cycle control (reviewed by McBride, 2013).

The limited number of proteins encoded within the 8 kb HPV genome forces an unusual economy; not only do most HPV proteins have multiple roles in the virus life cycle, but associations between HPV proteins increase the functional significance of the differentiation-dependent timing of viral protein expression. Previous work has demonstrated an association between E2 and the early proteins E1, E6 and E7. Interaction with E1 is important for viral genome replication (Mohr *et al.*, 1990), while association with E7 inhibits E7-dependent transformation (Gammoh *et al.*, 2006). The interaction between E2 and E6 causes relocalization of both proteins to nuclear speckles and a corresponding decrease in E2-dependent virus replication. E6 function is also affected by co-expression of E2, as demonstrated by a reduction in degradation of target proteins (Grm *et al.*, 2005). Concomitant with activation of late gene expression in the virus life cycle, E2 protein accumulates (Xue *et al.*, 2010). This is thought to result in repression of the E6- and E7-encoding early transcripts, promoting cellular differentiation and production of late transcripts (Bouvard *et al.*, 1994; Steger & Corbach, 1997; Thierry & Yaniv, 1987). E2 also forms a complex with E1^E4 and L2, which are expressed in the late stages of the life cycle. Interaction with E1^E4 stabilizes E2, potentially contributing to E2 protein accumulation (Davy *et al.*, 2009). Association of E2 and the minor capsid protein L2 facilitates recruitment of E2 to ND10 bodies (also known as PML-oncogenic domains) within the nucleus (Day *et al.*, 1998) and repression of E2-dependent transcription (Heino *et al.*, 2000). It is hypothesized that interaction of E2 with L2 is important for virus genome encapsidation.

At the end of the life cycle, newly synthesized L1 protein is imported into the nucleus as pentameric capsomeres through the karyopherin *α*2*β*1 heterodimer, where virus assembly takes place (Bird *et al.*, 2008; Merle *et al.*, 1999). The mature virion contains the major capsid protein, L1, and the minor protein, L2. L1 and L2 interact and, although the binding interface between L1 and L2 is poorly defined, studies indicate that L2 binds to hydrophobic residues of L1 and is therefore positioned in the central cavity of L1 pentamers (Finnen *et al.*, 2003; Lowe *et al.*, 2008). Sequences at the C terminus of L1 are important for non-specific interaction with DNA (Li *et al.*, 1997) and it has been suggested that key features in the L1 protein resemble histone chaperone proteins and that L1 may bind histones to facilitate genome packaging (Buck *et al.*, 2013).

In this study, we hypothesized that E2 associates with the major capsid protein, L1, and that this interaction is important in regulating E2 function in the late stages of the virus life cycle. We have confirmed this interaction in human keratinocytes and show for the first time that L1 co-operates with E2 to enhance transcription activation and virus DNA replication. Furthermore, we demonstrate that L1 affects the localization of E2 protein and that L1 is partially recruited to E1/E2 viral replication foci. We hypothesize therefore that L1 contributes to the regulation of E2 in the virus life cycle.

## Results

### *In silico* analysis of E2 and L1 interaction

**Fig. 1.**
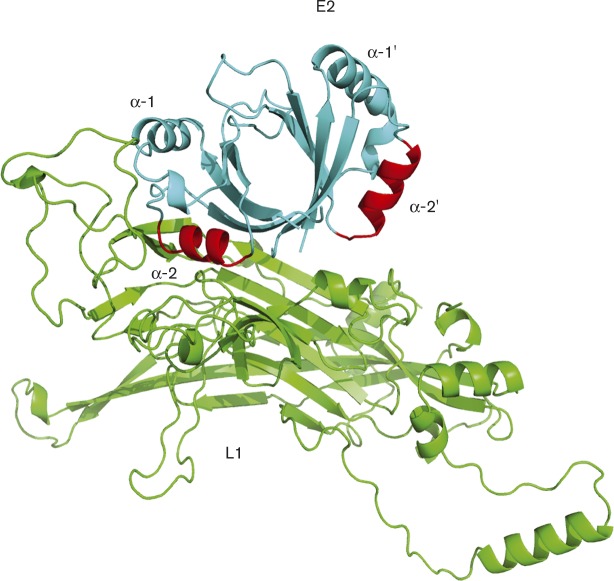
*In silico* analysis of potential binding interfaces between HPV16 E2 and L1. *In silico* modelling was performed using zdock, ClusPro and Rosetta. Monomeric L1 protein is shown in green and the homodimeric DNA-binding domain of E2 (aa 285–365) in cyan with the region of the α-2 helices (α-2 and α-2′) of E2 docked within 4 Å of L1 shown in red. The binding interface with the lowest energy was between an α-2 helix of E2 (aa 335–365) in close proximity to external loops between aa 250–290 of the L1 protein.

The interaction between E2 and L1 was computationally analysed and the best-docked complexes were obtained based on energy minimization. These complexes were further analysed to determine the residues that were in close proximity at the predicted binding interface (Fig. 1).

Based on consensus from 21 best-docked complexes, it was concluded that aa 335–365 of E2 have a high likelihood of association with L1. These residues make up the *α*-2 helix of the E2 DBD. The region of L1 predicted to bind to E2 was within an external loop between aa 250 and aa 290, which is also predicted to bind L2 (Lowe *et al.*, 2008).

### Characterization of the interaction between HPV16 E2 and L1

**Fig. 2.**
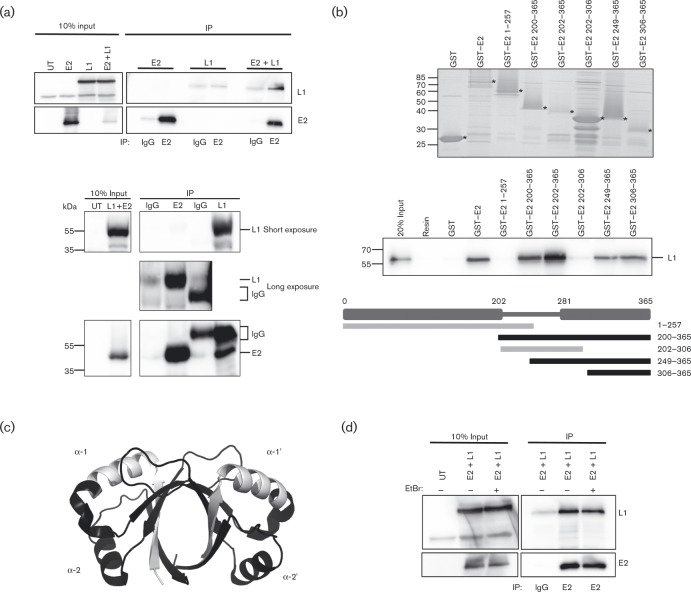
Characterization of the HPV16 E2 and L1 interaction. (a) Upper panel: C33a cells were transfected with E2 and L1 expression constructs alone or in combination, and lysates were immunoprecipitated (IP) with sheep anti-E2 or pre-immune IgG control. Co-immunoprecipitated protein complexes were separated by SDS-PAGE and L1 and E2 proteins were detected by Western blotting with mouse anti-L1 and anti-E2 (TVG261) antibodies. On the left, 10 % input of cell lysates is shown. Lower panel: C33a cells were transfected with E2 and L1 expression constructs, and lysates were immunoprecipitated with sheep anti-E2 or mouse anti-L1 antibodies and isotype-matched IgG control. Co-immunoprecipitated protein complexes were separated by SDS-PAGE, and L1 and E2 proteins were detected by Western blotting with mouse anti-L1 and anti-E2 (TVG261) antibodies. On the left, 10 % input of cell lysates is shown. A short and a long exposure of the L1 blot are shown to allow visualization of the protein precipitated with L1- and E2-specific antibodies. (b) GST pull-down assays using GST, GST-E2 full-length and the truncations indicated in each lane. Upper panel shows a Coomassie-stained gel of purified GST-E2 proteins, each indicated by *. Middle panels show bound purified L1 capsomeres detected by Western blotting. On the left, 20 % input is shown. This experiment was performed three times and similar results were obtained on each occasion. A representative Western blot is shown. The scheme in the lower panel represents relative binding affinity of each E2 truncation. Those that bound L1 are shown in black, those that did not bind are shown in grey. (c) Three-dimensional image of the dimerized DNA-binding domain of E2 obtained from PDB. The areas shown in black depict the regions of E2 that are important for the interaction with L1. (d) Co-immunoprecipitation assays as described in (a) were performed in the presence of ethidium bromide (EtBr) to disrupt DNA–protein interactions.

To determine whether E2 associates with L1 in a cell-based assay, co-immunoprecipitation experiments were carried out. HPV-negative cervical carcinoma-derived C33a cells were co-transfected with HPV16 E2 and codon-optimized L1 expression constructs alone or in combination, and the proteins immunoprecipitated with E2-specific antibody or isotype control (IgG). L1 protein was robustly co-immunoprecipitated with E2-specifc antibody when co-expressed with E2 and only a minimal amount of L1 protein was non-specifically immunoprecipiated in the absence of E2 protein, demonstrating specificity of the assay (Fig. 2a). We also performed the reverse co-immunoprecipitation, where complexes were immunoprecipitated with L1 protein-specific antibody and co-precipitated E2 was detected by Western blotting (Fig. 2a).

To further test our *in silico* analysis of the E2–L1 binding surfaces, domain mapping experiments using full-length and truncated glutathione-*S*-transferase (GST)-tagged E2 proteins were carried out. GST-tagged E2 proteins bound to glutathione-agarose beads were incubated with purified L1 capsomeres. As expected, L1 capsomeres bound with high affinity to full-length E2 while no binding was observed to the affinity resin alone or the GST-bound resin (Fig. 2b). Domain mapping revealed that the L1 binding site within E2 exists in the C-terminal transactivation domain as aa 202–365 bound L1 capsomeres, while aa 1–257 did not. Furthermore, aa 306–365 at the extreme C-terminal region of E2 bound L1 protein, while aa 202–306 were unable to bind L1. The L1-binding region between aa 306 and aa 365 is outside the DNA-binding surface of E2 (*α*-1 helices) but contains the *α*-2 helices that were predicted to bind L1 in our *in silico* analysis (Figs 1 and 2c), providing evidence that the binding model may reflect the true binding interface. These binding experiments demonstrate a previously uncharacterized direct interaction between HPV16 E2 and L1 proteins that could have important implications for the virus life cycle.

As both L1 and E2 proteins are known to bind to DNA (Dell *et al.*, 2003; Schäfer *et al.*, 2002), and the L1 binding surface on E2 was within the C-terminal DNA-binding domain but outside the specific DNA-binding helices of E2, it is possible that the interaction is mediated by both proteins simultaneously associating with contaminating DNA. We therefore repeated co-immunoprecipitation experiments in the presence of ethidium bromide, which intercalates with DNA and prevents protein–DNA interactions. Incubation of the protein lysates with ethidium bromide did not affect the ability of E2 and L1 to form a complex (Fig. 2d). It is therefore unlikely that this novel protein–protein interaction is mediated by interaction with DNA.

### L1 stimulates E2-dependent transcription activation and replication

**Fig. 3.**
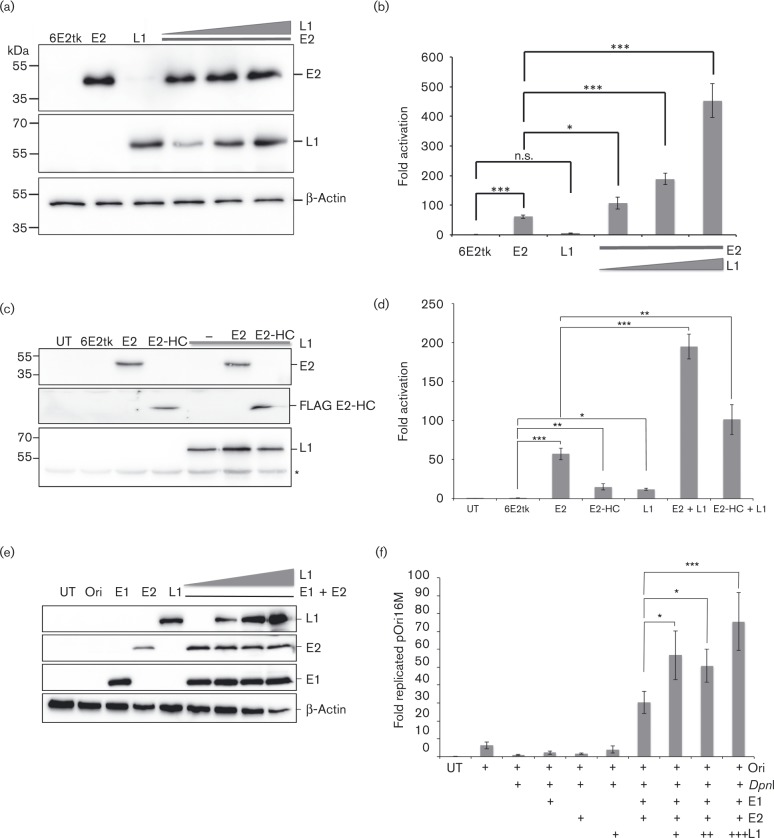
L1 co-expression increases E2-dependent transcription activation. (a) C33a cells were co-transfected with an E2-responsive firefly luciferase reporter (6E2tk), E2 expression plasmid, and increasing amounts of L1 expression plasmid. E2 and L1 protein levels were determined by Western blotting. Molecular mass standards are indicated on the left. (b) Luciferase activity in cell lysates was measured and normalized to activity in cells transfected with 6E2-tk-Luc alone. (c) C33a cells were co-transfected as described in (a) and (d) transcription activity of full-length E2 was compared with a truncated E2 protein containing aa 200–365 (E2-HC). (d) Luciferase activity was determined as described in (b). (e) C33a cells were co-transfected with HPV16 Ori plasmid, E2 and E1 expression plasmids and increasing amounts of L1 expression plasmid. Protein expression was detected by Western blotting. Molecular mass standards are indicated on the left. (f) *Dpn*I-digested DNA quantified by qPCR. The data are expressed as fold increase in replication over *Dpn*I-digested pOri16M alone. All data shown represent the mean ± se of three independent experiments. Significance was tested using Student's *t*-test; *P < 0.05, **P < 0.01, ***P < 0.001.

To determine whether the association between HPV16 E2 and L1 alters E2 function, the ability of E2 to activate transcription in the presence of L1 was analysed. C33a cells were transfected with a luciferase reporter plasmid containing six E2 binding sites upstream of a thymidine kinase promoter, which controls expression of firefly luciferase, along with full-length E2 and increasing amounts of L1 plasmid. Protein expression was confirmed by Western blotting (Fig. 3a). Expression of E2 alone resulted in an over 50-fold activation of transcription from the reporter plasmid (Fig. 3b). Expression of L1 protein alone resulted in a small increase in transcription in comparison with reporter alone, but this did not reach significance (*P* = 0.06). Interestingly, co-expression of L1 with E2 resulted in a dose-dependent increase in luciferase activity, indicating that E2 and L1 co-operate to stimulate transcriptional activation.

The binding assays shown in Fig. 2b demonstrated that L1 protein associates with the C-terminal DNA-binding domain of E2. We therefore repeated the transcription assay using the truncated E2 protein E2-HC, which contains aa 200–365 of HPV16 E2. This E2 protein is able to bind to DNA and to L1 protein (Fig. 2b), but does not contain the transactivation domain of E2 and therefore should be severely crippled in its ability to activate the E2-responsive reporter. Surprisingly, this truncated E2 protein was able to activate transcription from the synthetic reporter, but activity was fivefold lower than that of full-length E2 (Fig. 3c and 3d). Co-transfection of L1 with E2-HC resulted in a further 10-fold activation of transcription, indicating that E2-dependent recruitment of L1 to the promoter is sufficient for transcriptional enhancement and that the inherent ability of E2 to function as a transcriptional activator is not necessarily required.

Using a transient DNA replication assay, the effect of L1 on E2-dependent virus replication was assessed. Cells were transfected with a plasmid containing the viral Ori and E1 or E2 alone or in combination, along with increasing amounts of the L1 expression plasmid. Protein expression was detected by Western blotting (Fig. 3e) and replicated Ori-containing plasmid was detected by quantitative PCR (qPCR) (Fig. 3f). As expected, co-expression of E1 and E2 resulted in a dramatic increase in Ori-dependent replication. This was enhanced by co-expression of L1 in a dose-dependent manner.

**Fig. 4.**
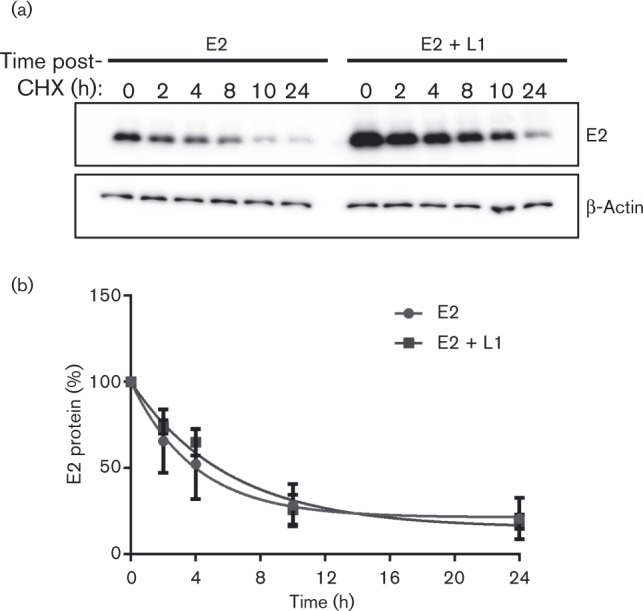
L1 does not alter E2 protein stability. C33a cells were transfected with E2 expression vector alone or in combination with L1. Cells were treated with cycloheximide (CHX) and harvested at the stated times. (a) E2 protein levels were determined by Western blotting and normalized to β-actin levels (detected on the same membrane). (b) Band intensities were measured using ImageJ. The data represent the mean ± se of three independent experiments.

Having demonstrated that the transcription and replication functions of E2 are stimulated by L1, it was important to determine whether these effects are due to alterations in E2 protein stability in the presence of L1. To determine the half-life of E2 alone or co-expressed with L1, C33a cells were transfected with E2 and L1 expression plasmids alone or in combination. Extracts of cells treated with cycloheximide for 0, 2, 4, 8, 10 and 24 h were analysed by Western blotting (Fig. 4a). E2 protein levels were normalized to *β*-actin protein and used to determine the half-life of E2 in each experimental condition (Fig. 4b). It should be noted that an increase in E2 protein expression was observed following co-expression with L1 protein. As expression of both of these proteins is driven from a CMV promoter, we presume that the increase in E2 protein when co-expressed with L1 is due to the enhancement of transcription activity of E2, which can enhance CMV promoter activity. This effect was not observed in the transcription assays shown in Fig. 3 because the amount of plasmid used in the transcription assays was the equivalent of 10-fold less than the amount used in the stability assays. The calculated half-life of E2 protein was 4.5 h, which is in agreement with previous reports (King *et al.*, 2011; Li *et al.*, 2014). A small increase in the half-life of E2 was observed in the presence of L1 (5.8 h), but this increase did not reach significance, suggesting that the effect of L1 on E2 activity is not due to stabilization of E2. Taken together, these data provide evidence that L1 directly alters both the transcriptional activation and replication functions of E2.

### L1 co-localizes with E2 and prevents nucleolar accumulation of E2

**Fig. 5.**
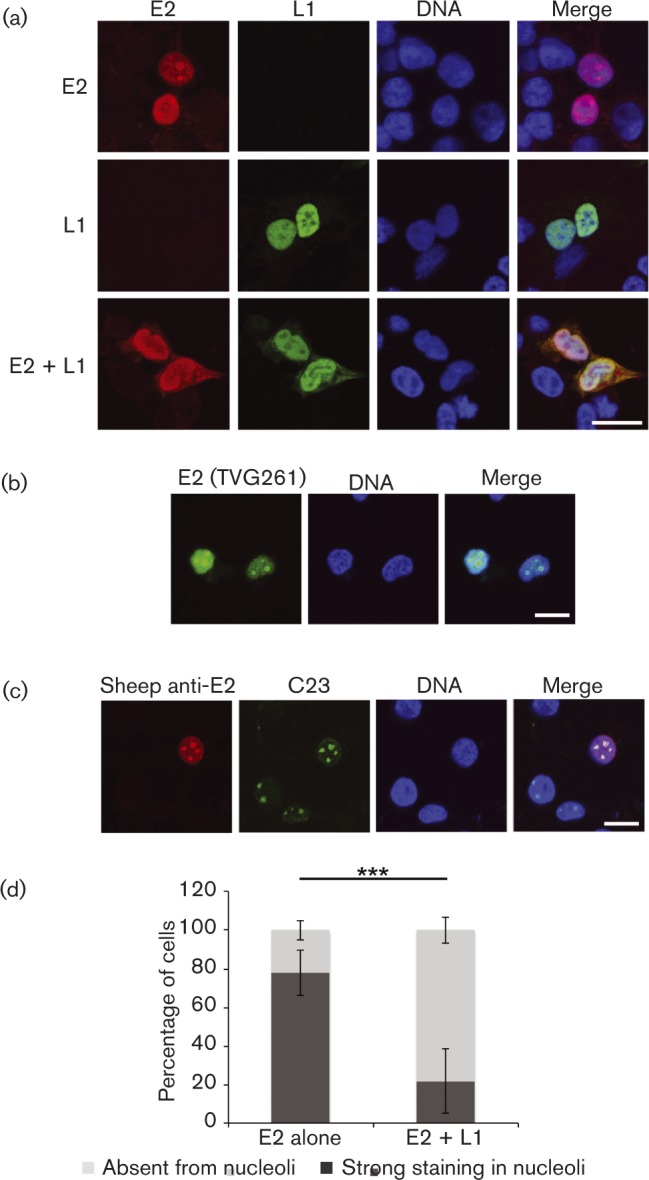
Subcellular localization of HPV16 E2 and L1. Confocal imaging. (a) C33a cells transfected with E2 and L1 expression plasmids alone or in combination. E2 protein was stained with sheep anti-E2 antibody (red) and L1 protein was stained with mouse anti-L1 antibody (green). DNA was stained with Hoechst (blue). (b) E2 protein was detected with mouse monoclonal anti-E2 antibody (TVG261; green). (c) E2 protein was detected with sheep anti-E2 antibody (red), and nucleolar marker protein C23 is shown in green. Bars (all panels), 10 μm. (d) E2-expressing cells transfected alone or in combination with L1 were scored for strong E2-staining in the nucleolus or E2-staining absent from the nucleolus. At least 80 cells were scored for each experiment. The data shown represent the mean ± se of three independent experiments. Significance was determined using Student's *t*-test; ***P < 0.001.

To further characterize the interaction between E2 and L1, subcellular localization of these two proteins expressed alone or in combination was investigated by confocal microscopy. When expressed alone, E2 protein was localized to the nucleus, and in many cells the staining of large foci within the nucleus was noted (Fig. 5a). These E2 foci have been previously reported for HPV1 E2 (Prescott *et al.*, 2014) and were shown to be nucleoli. It has also been demonstrated that treatment of cells with 100 mM salt prior to fixation results in strong nucleolar localization of HPV16 E2 (Sakakibara *et al.*, 2013). To confirm that the nuclear structures stained with our E2 antibody were nucleoli, we co-stained E2-transfected cells with an antibody specific for a nucleolar marker, C23. Co-localization of E2 with C23 was observed, demonstrating that E2 localizes to nucleoli in a subset of transfected cells (Fig. 5b). Furthermore, we stained cells with a commercially available mouse monoclonal HPV16 E2-specific antibody and confirmed the presence of E2-positive nucleolar staining, demonstrating that the staining observed with the sheep polyclonal HPV16 E2 antibody was reproducible (Fig. 5c). When expressed alone, HPV16 L1 was also observed in the nucleus, but with nucleolar exclusion in the majority of cells imaged. Of particular note, when E2 and L1 were co-expressed, the proteins co-localized in the nucleus and the proportion of cells with nucleolar E2-staining was significantly reduced (Figs. 5a, d).

**Fig. 6.**
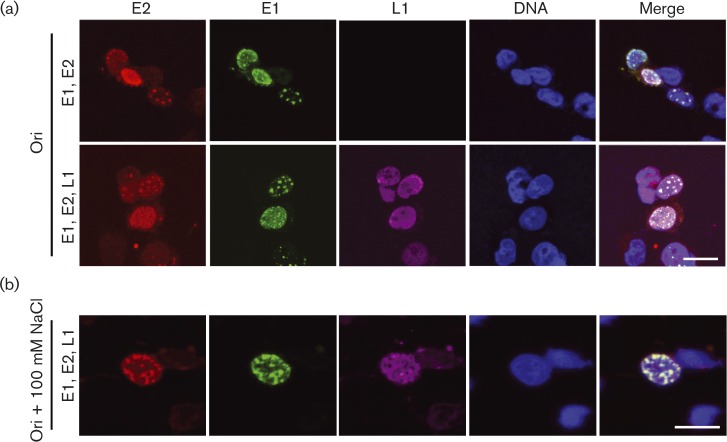
L1 protein is recruited to E1/E2 replication factories. (a) C33a cells were transfected with the Ori-containing plasmid, E1 and E2 (upper panel), and co-transfected with an L1 expression plasmid (lower panel). E2 protein was stained with sheep anti-E2 antibody (red), E1 with rabbit anti-HA antibody (green) and L1 with mouse anti-L1 antibody (magenta). DNA was stained with Hoechst (blue). (b) Cells were transfected as described for (a) and extracted with 100 mM NaCl prior to fixation as described in Methods. Images were captured by confocal microscopy. Bars, 10 μm.

E1- and E2-dependent virus genome replication has been shown to occur in discrete replication factories that form nuclear foci (Sakakibara *et al.*, 2011, 2013). Owing to its ability to enhance E2-dependent replication, the localization of L1 protein to E1/E2 replication foci was studied. As previously described, when co-transfected with the viral Ori-containing plasmid, E1 and E2 co-localized in bright foci, which are thought to be replication factories (Sakakibara *et al.*, 2013) and are distinct from the nucleolar staining described above. Co-transfection of L1 did not appear to alter the formation of E1/E2-associated replication foci and L1 was not enriched within the replication foci when cells were fixed in formaldehyde (Fig. 6a). However, extraction of the cells with buffer containing 100 mM NaCl prior to fixation revealed that a proportion of L1 protein co-localizes with E1/E2 replication foci, indicating that L1 may stimulate E2-dependent replication by recruitment to replication factories (Fig. 6b).

### Co-localization of E2 and L1 in differentiating epithelium

**Fig. 7.**
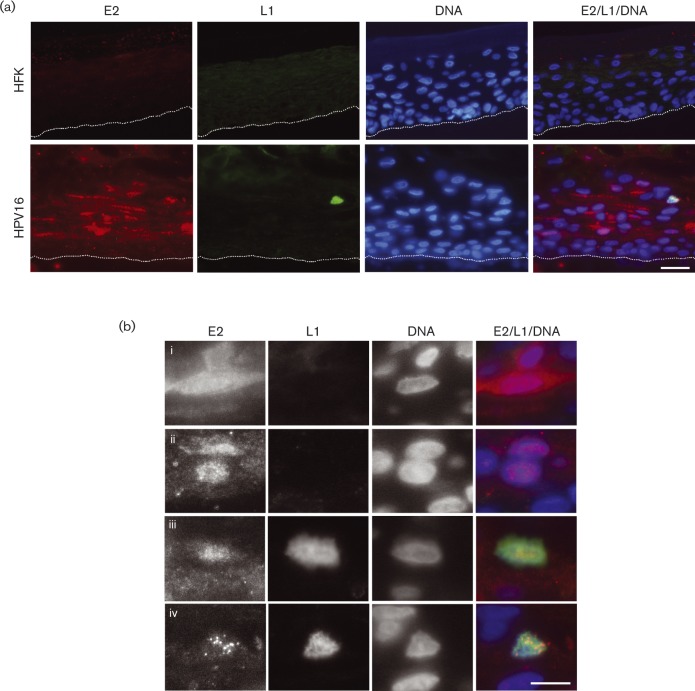
L1 and E2 co-localize in differentiating epithelium. (a) Sections of organotypic raft cultures derived from primary HFKs or HPV16-genome-containing HFKs from the same donor were stained with rabbit anti-E2 antibody (Xue *et al.*, 2010) (red) and mouse anti-L1 antibody (green) and DNA-stained with Hoechst (blue). The basal cells in each panel are highlighted by the dotted line. Bar, 10 μm. (b) Enlarged images highlight E2-positive, L1-negative cells with strong cytoplasmic and nuclear staining (i) and nuclear staining (ii). L1- and E2-positive cells show nuclear L1- and E2-staining (iii) and nuclear L1-staining with distinct E2 foci (iv). Bar, 5 μm.

To determine whether the interaction between E2 and L1 is physiologically relevant, the co-localization of these two proteins was studied in primary human foreskin keratinocytes (HFKs) containing HPV16 episomes. Thirteen-day-old organotypic rafts were fixed and sections co-stained with E2 and L1-specific antibodies. E2 staining was observed in the nucleus and cytoplasm of cells in the middle and upper layers of the raft section (Fig. 7a). While some E2-positive cells were stained in both the nuclear and cytoplasmic compartments [Fig. 7b(i)], some cells had E2 expression that was predominantly localized to the nucleus with no obvious cytoplasmic staining [Fig. 7b(ii)]. As described previously (Xue *et al.*, 2010), E2 staining in the basal and lower layers of the raft was not observed, presumably because the amount of E2 protein expressed in these cells is below the level of detection (Fig. 7a). No staining was observed in organotypic raft sections derived from HPV-negative HFKs, demonstrating specificity of the antibody. Of note, nuclear L1 staining was observed in individual cells in upper layers of the raft sections. The nuclei of these cells also stained positive for E2 [Fig. 7b(iii, iv)]. Some of the L1-positive cells contained distinct E2 foci within the nuclear compartment that were not observed in L1-negative cells, indicating that E2 may have a different biological function in differentiated L1-expressing cells. These data provide evidence that E2 and L1 are co-expressed in a physiologically relevant model system and that the physical association of these two viral proteins is likely to play a role in the HPV life cycle.

## Discussion

We predicted that the HPV replication and transcription factor E2 forms a complex with the late viral capsid protein L1. We initially tested this hypothesis by mapping potential interacting surfaces using protein–protein docking algorithms. These studies predicted that the *α*-2 helices of the C-terminal DNA-binding domain of E2 associate with L1 loops that lie on the surface of the capsomere knobs. The position of the predicted binding surface on L1 implies that E2 could bind to L1 when assembled as a pentameric capsomere, since the loops predicted to bind to E2 protrude from the surface of the capsomere knobs (Modis *et al.*, 2002). Interestingly, the region of L1 predicted to associate with E2 overlaps with the L2 binding region predicted by Lowe *et al.* (2008), although since L1 protein is pentameric in solution, it is possible that L1–L2–E2 complexes could exist. The L1 binding surface within E2 was predicted to lie within the *α*-2 helices of the E2 DNA-binding domain, which is outside the DNA-binding region contained within the *α*-1 helices, and was confirmed by domain-mapping experiments that showed that aa 306–365 of E2 were sufficient for L1 binding. This specific location of the binding site on E2 suggests that E2 may be able to bind to L1 and DNA simultaneously. Although the exact binding interface between L1 and E2 needs further refinement, an interaction was confirmed by co-immunoprecipitation experiments in human cervical keratinocytes. In addition, we showed that the interaction is not mediated by association with DNA. Interestingly, E2 and L1 were co-distributed in a subset of cells in the upper layers of differentiating epithelium in a physiologically relevant HPV16 life cycle model system.

Characterization of the effect of L1 on E2 activity revealed that L1 stimulates E2-dependent replication and transcription activation, without altering E2 protein stability. Although the effect of L1 overexpression on E2-dependent replication was statistically significant, only a 2.5-fold increase in replication was observed. It is possible that this increased replication is due to alteration of cell cycle progression of transfected cells, although changes in cellular proliferation were not noted. It is also possible that the effect of L1 on E2 activity is enhanced in differentiated cells, a possibility we are currently testing.

Co-expression of L1 altered the subcellular localization of E2. When E2 was expressed alone, E2-specific nucleolar staining was observed in many cells. The localization of E2 at the nucleolus has not been widely reported, but has been previously observed in a range of human cell types in our own immunofluorescence experiments (J. L. Parish, unpublished) and reported by others for HPV1 E2, or for HPV16 E2 following salt extraction of cells (Prescott *et al.*, 2014; Sakakibara *et al.*, 2013). The function of E2 at the nucleolus is not known, but it is interesting to note that the Epstein–Barr virus nuclear antigen 1 (EBNA1) protein, which is functionally analogous to E2, is also recruited to the nucleolus (Shire *et al.*, 2006). Furthermore, it has been shown that mutation of dipeptide recognition motifs of serine-arginine protein kinase 1 (SRPK1) in the hinge region of HPV1 E2 significantly increases enrichment of E2 at the nucleolus. HPV1 E1^E4 inhibits SRPK1 activity and enhances nucleolar localization of E2 (Prescott *et al.*, 2014). Although HPV16 E2 does not contain SRPK1 recognition motifs, it is tempting to speculate that, in the context of the virus life cycle, the temporal expression of E1^E4 and L1 could regulate nucleolar localization of E2 as cells terminally differentiate. Further work is required to understand the function and control of E2 at the nucleolus, but emerging evidence suggests an interplay between viral and host cell proteins in the regulation of E2 localization and activity. Perhaps of greater interest is the observation that L1 protein partially co-localized with E1/E2 replication factories following salt extraction of cells. These experiments indicate that a pool of L1 protein is recruited to replication factories, presumably to enhance virus replication.

**Fig. 8.**
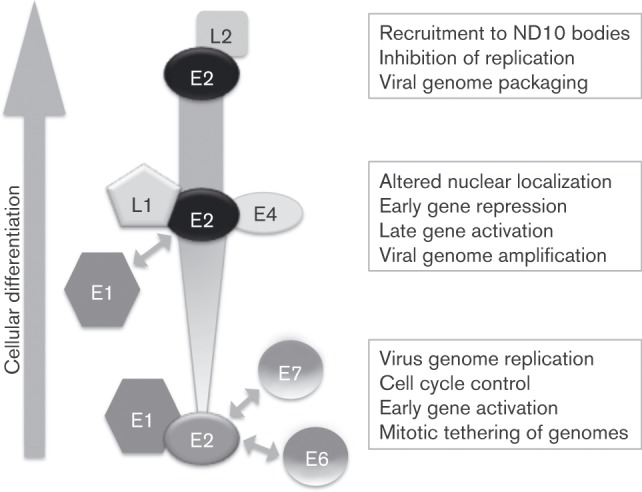
Model of differentiation-dependent E2 regulation via interaction with early and late HPV proteins. E2 protein associates with E1, E7, E1^E4 (E4), L1 and L2, the details of which are described in the text. As cells differentiate, E2 protein expression is increased. The functional consequence of interaction of E2 with other viral proteins is indicated.

The mechanistic regulation of E2 activity in the context of the virus life cycle is not understood. However, characterization of the interaction with L1 and other viral proteins demonstrates the ability of the virus to self-regulate E2 activity in differentiating epithelium (Fig. 8). Association of E2 with E1 in the basal cells is important for viral genome replication (Sanders & Stenlund, 2001), while association with the E6 and E7 proteins appears to regulate oncoprotein activity (Gammoh *et al.*, 2006; Grm *et al.*, 2005). In addition, E6 appears to inhibit E2-dependent virus replication (Grm *et al.*, 2005). Differentiation-induced expression of E1^E4 in the mid-layers of the epithelium results in a stabilization of E2 (Davy *et al.*, 2009), which is consistent with the observed increased expression of E2 in the mid- and upper layers of cervical epithelia (Xue *et al.*, 2010) and organotypic raft cultures of HPV16-positive primary HFKs (Fig. 7). Whether E2 is able to simultaneously interact with E1^E4 and L1 in differentiated cells remains to be determined.

Our data show that L1 enhances E2-dependent virus replication and, following salt extraction of cells, we observed that L1 protein co-localizes with E1/E2 replication foci in the presence of viral origin of replication. Put together with the observation that E2 and L1 proteins are co-expressed in HPV16-genome-containing differentiating epithelium, it is possible that L1 contributes to E2-dependent viral genome amplification in the late stages of the virus life cycle. We also demonstrate that L1 co-expression increases transcription from an E2-responsive reporter. This is somewhat surprising since E2 is thought to switch from a transcriptional activator to a repressor as E2 levels rise in differentiating epithelium, resulting in repression of the E6 and E7 oncoproteins and subsequent cell cycle exit (Thierry, 2009). However, our experiments were performed using a synthetic E2-responsive promoter, which is a useful assay in the measurement of E2 activity in transfected cell systems such as that used in this study, but does not truly reflect the function of E2 as a transcriptional regulator at the viral long control region in differentiating epithelium. It is also conceivable that E2 and L1 do not act synergistically on the synthetic promoter and that the enhancement of transcription observed is not a direct result of the E2–L1 protein–protein interaction but an additive effect of independent promoter stimulation by E2 and L1. Nonetheless, the enhancement of E2 activity by L1 is intriguing and is likely to contribute to the self-regulation of E2 throughout the virus life cycle. It is also possible that the interaction between L1 and E2 does not regulate E2 activity in the context of the HPV life cycle, but plays a role in an as yet understudied step in the life cycle, such as the formation of virus particles. Finally, in the upper layers of the epithelium, L2 expression is initiated, which inhibits E2-dependent replication and facilitates virus genome packaging (Day *et al.*, 1998).

In summary, the differential regulation of E2 activity by early and late viral proteins appears to allow the use of a single viral protein to support multiple important life cycle events. Our data provide evidence of an interaction between E2 and the capsid protein L1, further adding to the complexity of E2 regulation during late stages of the virus life cycle.

## Methods

### *In silico* protein interaction analysis

Potential interactions between HPV16 L1 and E2 were computationally analysed using multiple-docking programs. The protein database (PDB) coordinate files for HPV16 L1 (1DZL) (Modis *et al.*, 2002) and HPV16 E2-DNA-binding domain (1ZZF) were used and protein–protein interactions were predicted using ZDOCK 3.0.2 (http://zdock.umassmed.edu) (Pierce *et al.*, 2014), ClusPro (http://cluspro.bu.edu) (Comeau *et al.*, 2004) and Rosetta (Lyskov & Gray, 2008).

### Cell culture

C33a (human cervical carcinoma epithelial) cells were grown in Dulbecco's modified Eagle's medium containing 10 % FBS at 37 °C, 5 % CO_2_, using standard tissue culture techniques. The transfection of primary HFKs from neonatal foreskin epithelia (ethical approval number 06/Q1702/45) was performed in Dr S. Roberts’ laboratory as previously described (Wilson *et al.*, 2007). A plasmid containing the wild-type HPV16 114 kb genome (obtained from Ethel-Michele de Villiers, DKFZ, Germany) was digested with *Bam*HI and recircularized and transfected into early-passage primary HFKs with a neomycin resistance marker. Twenty-four hours later, cells were seeded onto irradiated J2-3T3 fibroblasts in the presence of G418 in E medium (Wilson & Laimins, 2005) for 8 days. Emerging colonies were pooled and the presence of episomal HPV16 genomes was determined by Southern blotting (C. D. James and S. Roberts, unpublished data).

Organotypic raft cultures were prepared as previously described (Wilson & Laimins, 2005) and cultured for 13 days. Rafts were fixed in 3.7 % paraformaldehyde and paraffin-embedded prior to sectioning (Propath).

### Plasmids

pUF3-16L1h expresses codon-optimized HPV16 L1 from a CMV promoter obtained from Martin Müller, DKFZ, Germany (Leder *et al.*, 2001). pJ4Ω16E2 expresses HPV16 E2 from a CMV promoter (Bouvard *et al.*, 1994). Haemagglutinin (HA)-tagged HPV16 E1 was expressed from plasmid pHPV16-E1HA, obtained from Mart Ustav, Estonain Biocentre, Estonia. p6E2-tk-Luc contains six E2 binding sites upstream of a thymidine kinase promoter which drives firefly luciferase expression (Vance *et al.*, 1999). pOri16M was obtained from Iain Morgan, University of Virginia, USA, and contains a modified HPV16 Ori (nt 7838–139) (Taylor & Morgan, 2003). GST-fused HPV16 E2 truncations were expressed from pGEX2T constructs obtained from Professor Lawrence Banks, ICGEB, Trieste (Gammoh *et al.*, 2006; Johansson *et al.*, 2012), with the exception of GST-E2 306–365, which was constructed by ligation of a PCR product encoding these amino acids into the *Bam*HI and *Eco*RI sites of pGEX4T-1.

### Antibodies

HPV16 E2 was detected with either affinity-purified sheep anti-HP V16 E2 antibody which was produced by immunization with bacterially expressed and purified hexahistidine-tagged HPV16 E2 N-terminal domain (aa 1–216; Dundee Cell Products), mouse anti-HPV16 E2 TVG261 (Abcam) or affinity-purified rabbit polyclonal anti-HPV16 E2 antibody (obtained from Françoise Thierry, Institute of Medical Biology, Singapore) (Xue *et al.*, 2010). L1 was detected with mouse anti-HPV16 L1 antibody (Abcam). Mouse anti-HA antibody 12CA5 (Sigma-Aldrich) and rabbit anti-HA antibody HA.11 (Abcam) were used to detect HA-tagged HPV16 E1. Non-specific rabbit and mouse IgGs were purchased from Santa Cruz Biotechnology. Mouse anti-*β*-actin was purchased from Sigma-Aldrich. Horseradish peroxidase-conjugated secondary antibodies were used for Western blotting (Thermo Scientific). Alexa-Fluor-conjugated secondary antibodies were used for immunofluorescence (Invitrogen).

### Co-immunoprecipitation

C33a cells (3 × 10^6^) were seeded into 10 cm dishes and transfected using X-tremeGENE 9 (Roche). Co-immunoprecipitations were performed as previously described (Parish *et al.*, 2006b).

### GST pull-down

GST fusion proteins were purified from bacteria induced with 0.5 M IPTG for 4 h at 37 °C. Bacteria were lysed (25 mM Tris/HCl pH 8.0, 250 mM NaCl, protease inhibitors, 5 mM DTT) and samples were sonicated for 30 s at 30 % amplitude. Cleared lysates were incubated with glutathione agarose slurry at 4 °C for 2–6 h with gentle agitation. The beads were washed in lysis buffer and bound proteins were separated by SDS-PAGE and Coomassie-stained. For binding assays, equal amounts of GST fusion proteins (assessed by SDS-PAGE) immobilized on resin were incubated with 50 ng CsCl-purified L1 capsomeres obtained from Professor Martin Muller, DKFZ, Germany (Thönes *et al.*, 2008). Binding buffer (200 μl; 50 mM Tris/HCl pH 7.4, 100 mM KCl, 0.1 mM EDTA, 0.2 % NP-40, 0.1 % BSA, 2.5 % glycerol) was added and the samples were incubated at 4 °C for 2 h with gentle agitation. Samples were washed extensively (100 mM Tris/HCl pH 7.4, 150 mM NaCl, 1 % NP-40, 200 mM KCl) and bound L1 protein was detected by Western blotting.

### Transcription assay

Transcription assays were performed as previously described (Feeney *et al.*, 2011). C33a cells (3 × 10^5^) were seeded into each well of a six-well plate and co-transfected with expression plasmids for E2 (50 ng) and L1 (100, 250, 500 ng) along with luciferase reporter plasmid p6E2-tk-Luc (100 ng). Firefly luciferase activity was determined by luciferase assay (Promega).

### Replication assay

C33a cells (2.5 × 10^5)^ were seeded into each well of a six-well plate and left to adhere overnight. Cells were then co-transfected with 25 ng pOri16M, expression plasmids for L1 (100, 250, 500 ng), E2 (10 ng) and E1 (600 ng). Salmon sperm DNA was included to normalize the DNA amount in each transfection. Forty-eight hours later, cells were washed in PBS and DNA extracted using 250 μl Hirt extraction buffer (0.6 % SDS, 10 mM EDTA). NaCl was added to a final concentration of 1.25 M, and samples were incubated overnight at 4 °C. The DNA was purified by phenol/chloroform/isoamyl alcohol extraction followed by ethanol precipitation, and the DNA pellet was resuspended in 20 μl water. DNA was digested with *Dpn*I at 37 °C for 4 h and replicated pOri16M was measured by qPCR using Sensimix SyBr (Bioline) and an MXPro3005 PCR machine (Agilent) as previously described (Taylor & Morgan, 2003).

### Immunofluorescence

C33a cells were seeded onto glass coverslips prior to transfection with Lipofectamine 2000 (Invitrogen). All subsequent incubations were performed at room temperature. Twenty-four hours post-transfection, cells were fixed with 4 % formaldehyde for 10 min and permeabilized in 0.2 % Triton X-100/PBS for 10 min. Immunofluorescent staining of HFK raft sections was performed following an agitated low-temperature antigen retrieval as previously described (Watson *et al.*, 2002). Cells were blocked (10 % heat-inactivated goat serum, 2 % BSA in PBS) for 1 h and stained as previously described (Parish *et al.*, 2006a). Epifluorescent imaging was performed on a Nikon E600 epifluorescent microscope fitted with a DXM1200F digital camera, and confocal images were captured on a Zeiss LSM 510 META confocal microscope.

### Protein stability assays

C33a cells (6 × 10^6^) were seeded in 15 cm dishes and transfected with X-TremeGENE 9 (Roche). Twenty-four hours later, cells were trypsinized, and counted, and 1 × 10^6^ cells were reseeded in 6 cm dishes. The following day, cells were treated with 10 μg ml^ − 1^ cycloheximide (Sigma) and harvested at 0, 2, 4, 8, 10 and 24 h, lysed in urea lysis buffer (50 mM Tris/HCl, pH 7.4, 9 M urea, 5 mM DTT) and sonicated for 15 s at 30 % amplitude. Protein concentration was quantified by Bradford assay and proteins detected by Western blotting. Relative amounts of proteins at each time point were quantified and normalized to *β*-actin using ImageJ software, and half-life was calculated using Graphpad Prism 4 software using a one-phase exponential decay model.
